# Early-Life Exposure to *Clostridium leptum* Causes Pulmonary Immunosuppression

**DOI:** 10.1371/journal.pone.0141717

**Published:** 2015-11-13

**Authors:** Fei Huang, Hong-mei Qiao, Jia-ning Yin, Yang Gao, Yang-hua Ju, Ya-nan Li

**Affiliations:** 1 Department of Orthopedics, China-Japan Union Hospital of Jilin University, Changchun, Jilin, PR China; 2 Department of Pediatrics, The First Hospital of Jilin University, Changchun, Jilin, PR China; 3 Department of Molecular Biology, Basic Medical College of Jilin University, Changchun, Jilin, PR China; Wayne State University, UNITED STATES

## Abstract

**Introduction:**

Low *Clostridium leptum* levels are a risk factor for the development of asthma. *C*. *leptum* deficiency exacerbates asthma; however, the impact of early-life *C*. *leptum* exposure on cesarean-delivered mice remains unclear. This study is to determine the effects of early-life *C*. *leptum* exposure on asthma development in infant mice.

**Methods:**

We exposed infant mice to *C*. *leptum* (fed-*CL*) and then induced asthma using the allergen ovalbumin (OVA).

**Results:**

Fed-*CL* increased regulatory T (Treg) cells in cesarean-delivered mice compared with vaginally delivered mice. Compared with OVA-exposed mice, mice exposed to *C*. *leptum* + OVA did not develop the typical asthma phenotype, which includes airway hyper-responsiveness, cell infiltration, and T helper cell subset (Th1, Th2, Th9, Th17) inflammation. Early-life *C*. *leptum* exposure induced an immunosuppressive environment in the lung concurrent with increased Treg cells, resulting in the inhibition of Th1, Th2, Th9, and Th17 cell responses.

**Conclusion:**

These findings demonstrate a mechanism whereby *C*. *leptum* exposure modulates adaptive immunity and leads to failure to develop asthma upon OVA sensitization later in life.

## Introduction

Asthma is a common, chronic respiratory disease that affects approximately 300 million people worldwide. It is prevalent in more than 10% of the population in many industrialized countries [1]. Asthma is characterized by airway inflammation and airway hyper-responsiveness (AHR); patients with asthma typically present with coughing, breathlessness, wheezing, and chest tightening [2]. T helper (Th)2 cells secrete the cytokines interleukin (IL)-4, IL-5, and IL-13, which play critical roles in the pathogenesis of asthma, stimulate immunoglobulin E production, and enhance eosinophil accumulation [3]. Notably, the cytokine milieu in which T lymphocytes are activated is one of the most critical elements in determining T helper differentiation. IL-25 and IL-33 were recently recognized as playing a central role in immune deviation toward pathogenic Th2 responses during the evolution of T helper effector cells [4,5]. Th1 secretions enhance neutrophilic infiltration, a hallmark of severe asthma, by inducing increased production of neutrophil-attracting chemokines in the lung [6,7]. Th17 cells are characterized by IL-17A, IL-17F, and IL-21 production; these cytokines also indirectly promote eosinophilic infiltration and airway macrophage survival by enhancing Th2 responses and B cell differentiation [8,9]. Increasing cellular immune network complexity, Th9 and Th22 cells are involved in the pathological and physiological changes of asthma. Th9 cells, which mainly secrete IL-9, are major contributors to the onset and progression of asthma inflammation; IL-9 is associated with mucus hypersecretion, bronchial hyperreactivity, and increased Th2 cytokine expression [10,11]. However, the exact mechanism of Th22 involvement in asthma development and pathogenesis has not been elucidated [4]. In contrast, regulatory T cells (Treg cells, a CD4^+^CD25^+^FOXP3^+^ subset) have inhibitory effects on asthma airway inflammation, inhibiting the proliferation of other T cells [12]; it is believed they are mediated by cytokines such as tumor growth factor beta (TGF-β) and IL-10 [13]. FOXP3 expression is considered a master regulator of Treg inhibition function [14]. FOXP3 associates with other transcription factors, including nuclear factor of activated T cells (NF-AT), augmenting Treg transcriptional machinery, cytokine production, and protein expression [15,16].

Most of the current literature suggests that the gastrointestinal tract of a normal fetus is sterile. During birth and rapidly thereafter, the infant’s gut is colonized by bacteria from the mother and the surrounding environment. It is obvious that exposure at birth would differ with the mode of delivery [17]. During vaginal delivery (VD), contact with the maternal vaginal and intestinal flora is an important source for initiating bacterial colonization of the infant’s gut. This direct contact is absent during cesarean delivery (CD), and non–maternally derived environmental bacteria play an important role in the intestinal colonization of the infant’s gut [18]. CD may alter gut bacterial flora, where VD newborns are colonized with beneficial bacterial strains earlier than CD newborns, whereas CD infants may have abnormal primary gut flora levels for up to six months after birth [19]. In addition, increased clostridia colonization is associated with CD [20], altering microbial stimuli that may influence immune system maturation and the development of antigenic tolerance, increasing the risk of asthma and other allergic diseases [21].

*Clostridium leptum* is one of the most dominant three bacteria in the human gut; in adult mice, it maintains the intestinal microecological balance, promotes immune maturation, and increases Treg numbers to alleviate airway inflammation [22,23]. We speculated that early-life exposure to *C*. *leptum* affects the development of allergen-induced pulmonary inflammation later in life.

In this study, we sought to determine the role of CD and early-life *C*. *leptum* feeding (fed-*CL*) on the development of allergic asthma (fed-*CL* before allergen sensitization and challenge). The question arose because of the lack of data on the impact of CD and fed-*CL* in infants and the lack of studies examining the effects of *C*. *leptum* on pulmonary inflammation in CD mice. We found that early-life fed-*CL* in CD infant mice created an immunosuppressive environment that increased CD4^+^CD25^+^FOXP3^+^ Treg numbers and impaired T effector cells, including Th1, Th2, Th9, and Th17 production and cytokine secretion *in vivo* following exposure to the allergen ovalbumin (OVA) after weaning. Taken together, our data suggest a mechanism for *C*. *leptum* exposure during infancy that alters pulmonary immune responses and affects asthma airway inflammation in the long term; nevertheless, the Treg increase was greater in CD mice than in VD mice following fed-*CL*.

## Methods

### *C*. *leptum* preparation

*C*. *leptum* was purchased from Jilin Baoxin Biological Technology (Changchun, China). We isolated the strain from a human fecal sample and stored it at −80°C. A single *C*. *leptum* colony was grown in chopped meat broth (before use, 500 μg/mL cycloserine and 15 μg/mL cefoxitin) for 24 h, harvested by centrifugation, and washed with phosphate-buffered saline (PBS). The freshly prepared bacteria in PBS were used for oral feeding.

### Preparation of CD mouse pups

Virgin female BALB/c mice (6–8 weeks old, 18–22 g) were obtained from the Jilin University Animal Research Center and housed in a specific pathogen–free (SPF) facility in a 12-h/12-h light/dark cycle with water and chow *ad libitum*. The mice were acclimated to the environment for three days; three mice were housed with a male BALB/c mouse overnight between 6:00 PM and 9:00 AM. Pregnancy was determined by the presence of a vaginal plug the next morning; this was designated Gestational Day 1. Pregnant mice were killed by cervical dislocation on Gestational Day 20, and caesarean section was performed to obtain the pups in a SPF barrier environment, and the pups were transferred to postpartum (24–48 h after vaginal delivery) female SPF mice that served as foster mothers. The experimental protocols were established according to the National Institutes of Health Animal Research and Care guidelines and were approved by the Jilin University ethics committee.

### The effect of early-life fed-CL on Treg frequency and number

We examined the effect of fed-*CL* on Treg frequency and number in CD suckling mice as compared with VD suckling mice. Eight-day-old CD and VD mouse pups were fed 10^8^ colonies of *C*. *leptum* in 100 μL PBS or PBS alone by gavage daily until weaned at 21 days old, and we analyzed the Treg frequency and number when the mice were 22 days old.

### Asthma model and grouping

**Fig 1:**
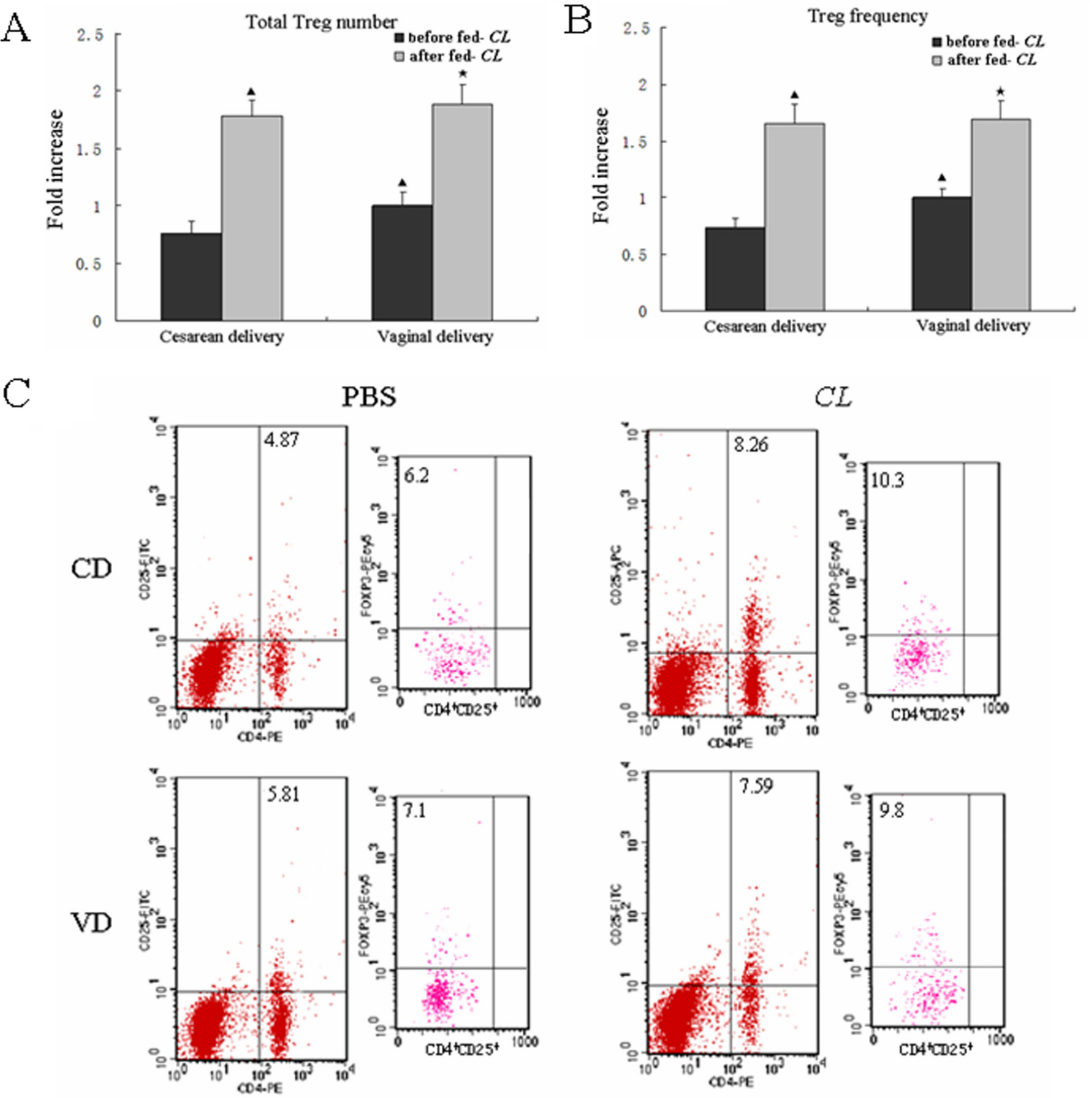
Fed-*CL* increased Treg numbers in CD mice as compared with VD mice. (A, B) Flow cytometry measurement of total MLN Treg numbers and frequency when mice were 22 days old; (C) the representative dot plots. Data are plotted as the means ± SD. ^▲^ *p* < 0.05 vs. CD mice before fed-*CL*; ^★^ *p* < 0.05 vs. VD mice before fed-*CL*.

To investigate the influence of fed-*CL* in a subsequent early-life model of OVA-induced asthma, we fed 8-day-old CD pups with *C*. *leptum* as described above before establishing an OVA model of asthma. There were 25 mice in each of the four CD test groups: *CL*-fed, OVA-sensitized (CD [C]/*CL*/OVA), vehicle-fed OVA-sensitized (C/-/OVA), *CL*-fed PBS-sensitized (C/*CL*/-), vehicle-fed PBS-sensitized (C/-/-) (control) (Fig 1). C/*CL*/OVA and C/-/OVA mice was sensitized by intraperitoneal injection with 10 μg OVA (grade V; Sigma-Aldrich, St. Louis, MO, USA) and 200 μg aluminum hydroxide in 100 μL PBS when they were 22 days old and boosted with the same reagent 14 days later. The control group of mice was injected with PBS. On days 50, 51, and 52, the mice were challenged with 1% aerosolized OVA or PBS for 20 min. On day 54, the mice underwent pulmonary function tests (PFT) and bronchoalveolar lavage, and the mediastinal lymph nodes (MLN) and lungs were collected for the experiments.

### CD4^+^CD25^+^ and CD4^+^CD25^–^ T cell isolation

CD4^+^CD25^–^ responder T cells were isolated from the MLN of 6–8-week-old untreated mice; CD4^+^CD25^+^ T cells were isolated from the MLN of fed-*CL* mice. Specifically, CD3^+^ T cells were purified from the MLN using a nylon wool column as previously described [24] and applied to a MACS Treg Isolation Kit (Miltenyi Biotec, Auburn, CA, USA). CD4^+^CD25^+^ and CD4^+^CD25^-^ T cell purity following magnetic bead depletion were both >95%; >95% of the CD4^+^CD25^+^ T cells were FOXP3^+^; <5% of the CD4^+^CD25^-^ T cells were FOXP3^+^. As >95% of the CD4^+^CD25^+^ T cells expressed FOXP3, we used magnetic bead depletion to isolate the CD4^+^CD25^+^FOXP3^+^ cells as Treg cells.

### Detection of induced Treg inhibitory activity

Enriched CD4^+^ T cells were sorted into the CD4^+^CD25^+^ Treg subset and the CD4^+^CD25^–^ responder T subset. The responder cells (2 × 10^5^) were plated in 96-well U-bottom plates along with the Treg cells at ratios of 1:1, 1:0.5, 1:0.25, and 1:0.125, or without any Treg cells (positive control), and cultured for 72 h at 37°C in 5% CO_2_. A [^3^H]-thymidine pulse (1 μCi/mL) was administered for the last 8 h of culture, after which proliferation was estimated by measuring the radioactivity incorporated. The inhibition efficiency (%) was calculated as follows: [(counts per minute [cpm] of positive control)—(cpm of experiment)/(cpm of positive control)] ×100.

### Flow cytometry analysis

Single-cell suspensions from the MLN were prepared and enriched by nylon wool. Referring to previous studies [25,26], we calculated the frequency of CD4^+^ CD25^+^FOXP3^+^ Treg cells in individual samples, as well as that of the effector T cells Th1 (CD3^+^CD8^–^interferon [IFN]-γ^+^), Th2 (CD3^+^CD8^–^IL-4^+^), Th9 (CD3^+^CD8^–^IL-9^+^), Th17 (CD3^+^CD8^–^IL-17A^+^), and Th22 (CD3^+^CD8^–^IL-22^+^IL-17A^–^). Cells were stained in PBS containing 1% fetal calf serum with the following antibodies (all from eBioscience): phycoerythrin (PE)-conjugated anti-CD4, fluorescein isothiocyanate (FITC)-conjugated anti-CD25, and PE-Cy5–conjugated anti-FOXP3; PE-conjugated anti-CD3, allophycocyanin-conjugated anti-CD8, FITC-conjugated anti–IFN-γ, anti–IL-4, anti–IL-9, anti–IL-17A, and PerCP-eFluor–conjugated anti–IL-22. Homotype-independent antibody was used as the negative control. IFN-γ, IL-4, IL-9, IL-17A, and IL-22 intracellular staining was performed after 4-h stimulation with 25 ng/mL phorbol 2-myristate 13-acetate, 1 mg/mL ionomycin, and 2 nmol/mL monensin (all from Sigma-Aldrich).

### Detection of *Foxp3* mRNA by real-time PCR (RT-PCR)

Total RNA was extracted from lung tissue using TRIzol reagent (Invitrogen, Carlsbad, CA, USA). Quantitative detection of the *Foxp3* and the internal reference mouse glyceraldehyde phosphate dehydrogenase (*Gapdh*) genes was carried out with SYBR Green–based quantitative RT-PCR with MMLV reverse transcriptase (Promega, Madison, WI, USA) and gene-specific primers. The primers used were as follows: *Foxp3*: (sense, S) 5′-CTTATCCGATGGGCCATCCTGGAAG-3′ and (antisense, A) 5′-TTCCAGGTGGCGGGGTGGTTTCTG-3′ (112-bp product); *Gapdh*: (S) 5′-GCACAGTCAAGGCCGAGAA-3′ and (A) 5′-CCTCACCCCATTTGATGTTAGTG-3′ (96-bp product). The RT thermal cycling reaction consisted of 70°C for 5 min, 42°C for 60 min, and 95°C for 5 min; the amplification thermal cycling reaction consisted of 40 cycles of 95°C for 30 s, 58°C for 30 s, and 72°C for 30 s. The results were analyzed using the comparative threshold cycle value (2^-ΔΔCT^) method [27].

### Enzyme-linked immunosorbent assay (ELISA) quantification of cytokine production

Referring to previous studies [28,29], the concentrations of secreted IL-4, IL-5, IL-13, IL-9, IL-22, IL-21, IL-23, IL-17A, IL-17F, IFN-γ, IL-10, TGF-β, IL-25, and IL-33 in the supernatants of the co-cultures were determined using the respective commercially available double-antibody sandwich ELISA kits (all from eBioscience).

### Airway resistance measurement

Dynamic lung resistance was measured using the flexiVent forced oscillation technique (SCIREQ, Montreal, Canada). The mice were anesthetized, ventilated, and airway lung resistance was measured in response to 0–50 mg/mL methacholine.

### Bronchoalveolar lavage

Two days after the final OVA challenge, each mouse underwent bronchoalveolar lavage with three aliquots of 250 μL PBS through a tracheal cannula. The collected bronchoalveolar lavage fluid (BALF) was centrifuged at 4°C at 600 ×*g* for 10 min, and the supernatant was stored at −70°C. The cells in each BALF sample were stained with trypan blue to determine the number of viable cells. Aliquots of BALF were applied to glass slides for Wright staining. Macrophage, eosinophil, lymphocyte, and neutrophil numbers in 200 cells per BALF sample were counted in a masked manner.

### Histological analysis and scoring

Lung tissues were fixed in 10% formalin overnight and paraffin-embedded. Tissue sections (5 μm) were stained with hematoxylin–eosin for histological assessment under light microscopy in a masked manner. The scoring system used has been described previously [30]: 0, no cells; 1, a few cells; 2, a ring of cells 1–cell layer deep; 3, a ring of cells 2–4-cell layers deep; and 4, a ring of cells >4–cell layers deep.

### Statistical analysis

All data are presented as the mean ± standard deviation (SD). We assessed differences between control and experimental groups by analysis of variance. We considered *p* < 0.05 to indicate statistical significance.

## Results

### Fed-*CL* increased Treg cells in CD mice compared with VD mice

We analyzed Treg frequency and number to examine the effect of fed-*CL* on Treg cells in mice delivered by different modes. Before fed-*CL* (when the mice were 8 days old), the Treg frequency and number in CD weanling mice were significantly lower than that in VD weanling mice. Continuous feeding with *C*. *leptum* for 14 days (i.e., up until the mice were 22 days old) increased Treg frequency and number more in CD weanling mice as compared to VD weanling mice (Fig 1); however, the difference was not significant.

### Construction of the asthma model and lung tissue collection

**Fig 2:**
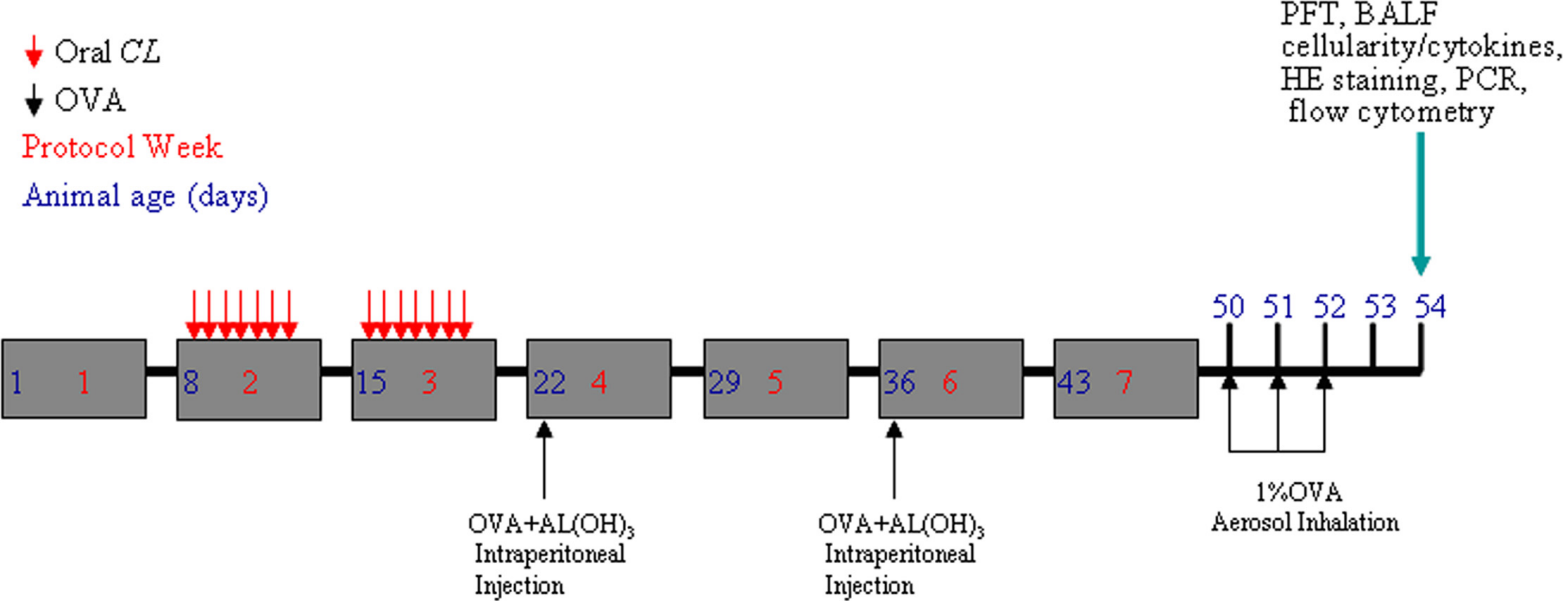
Schematic of a mouse model of early-life *C*. *leptum* ± OVA asthma. All analyses were carried out 48 h after the final protocol day. HE, hematoxylin–eosin; AL(OH)_3_, aluminum hydroxide.

Eight-day-old CD pups were fed with *C*. *leptum* as described above before we established an OVA model of asthma. The asthma model was established when the mice were 52 days old, and lung tissue samples were collected when the mice were 54 days old (Fig 2).

### Early-life fed-*CL* reduced airway responsiveness and cell numbers in BALF of CD asthmatic mice

**Fig 3:**
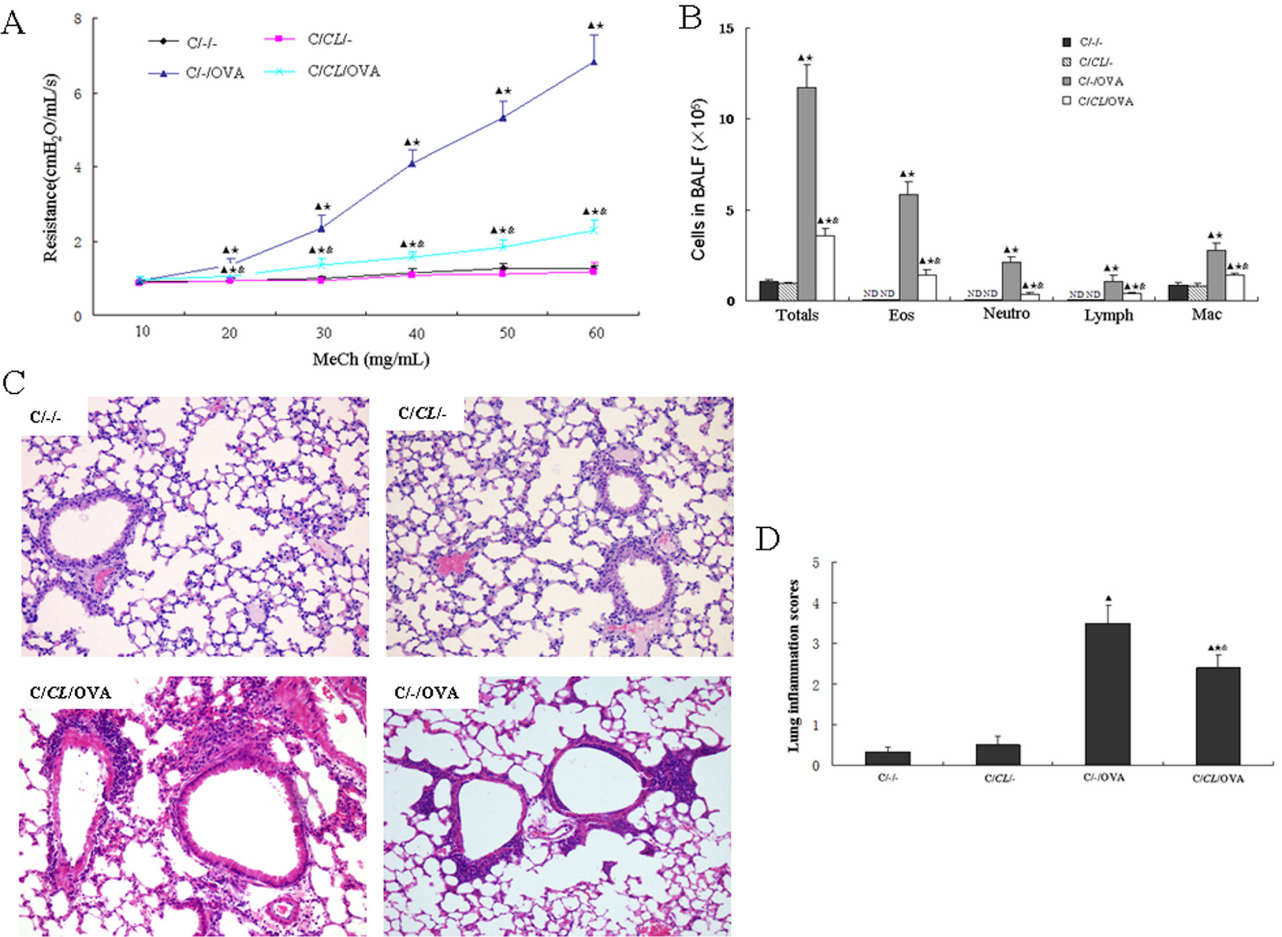
Early-life fed-*CL* reduced airway responsiveness, BALF cell numbers, and inflammatory cell infiltration in CD asthma mice. (A) AHR determined by airway resistance in response to inhaled methacholine (MeCh). (B) Total cell numbers and differential eosinophil (Eos), neutrophil (Neutro), lymphocyte (Lymph), and macrophage (Mac) counts in BALF. Lung tissue sections were stained with hematoxylin–eosin to evaluate allergic inflammation. (C) Representative images of lung tissue sections (original magnification, ×400). (D) Scoring of the extent of inflammation via quantitative analysis of inflammatory cell infiltration in lung sections. Data are plotted as the means ± SD. ^▲^ *p* < 0.05 vs. C/-/-; ^★^ *p* < 0.05 vs. C/*CL*/-; ^&^ *p* < 0.05 vs. C/-/OVA.

To investigate the effect of fed-*CL* in asthmatic infant mice, we fed 8-day-old CD mouse pups with *C*. *leptum* and established an asthma model after they had been weaned (Fig 2), and analyzed the pulmonary function 48 h after the final OVA challenge day. Resistance to increasing concentrations of methacholine gradually increased in the C/-/OVA and C/*CL*/OVA groups; resistance in both groups was significantly higher than that in the C/-/- group (Fig 3A). In contrast, resistance of the C/*CL*/- group was similar to that of the C/-/- group; resistance of the C/*CL*/OVA group was significantly reduced as compared to the C/-/OVA group (Fig 3A). Hence, oral feeding with *C*. *leptum* attenuates allergic airway resistance in asthmatic mice. Following the lung function tests, we collected and analyzed the BALF for cellularity. Total cell number, eosinophils, neutrophils, lymphocytes, and macrophages were significantly increased in the C/-/OVA mice (Fig 3B). In the C/*CL*/OVA group, the same cells were all noticeably decreased as compared with that in the C/-/OVA group, and their numbers were higher than that in the C/-/- and C/*CL*/- groups (Fig 3B). We further examined the effect of oral administration of *C*. *leptum* on allergic lung inflammation in mice, examining lung tissue sections from individual mice under hematoxylin–eosin staining. We observed airway epithelial hyperemia and edema, many pulmonary goblet cells, and inflammatory infiltrates in the lungs of the C/-/OVA mice, but not in the C/-/- and C/*CL*/- mice (Fig 3C and 3D). There was an obviously lower degree of airway epithelial hyperemia and edema, fewer pulmonary goblet cells, and inflammatory infiltrates in the lungs of the C/*CL*/OVA mice (Fig 3C and 3D).

### Early-life fed-*CL* induced Treg cells in CD asthma mice

**Fig 4:**
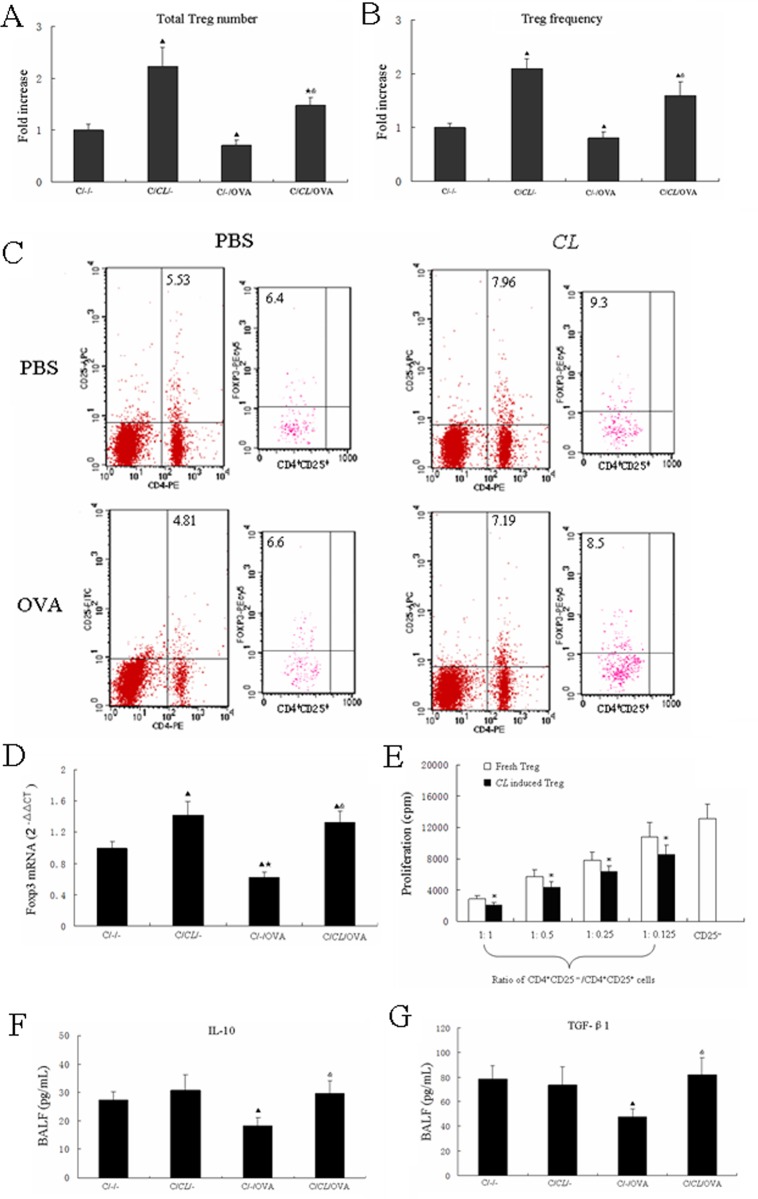
Early-life fed-*CL* increased Treg number and frequency. (A) Total numbers and (B) frequency of Treg cells in the CD mouse early-life asthma model were measured by flow cytometry and (C) representative flow dot plots. (D) *Foxp3* gene expression in whole lung tissue. (E) *In vitro* assay measurement of Treg inhibitory function. (F, G) ELISA detection of IL-10 and TGF-β1 in BALF. Data are plotted as the means ± SD. ^▲^ *p* < 0.05 vs. C/-/-; ^★^ *p* < 0.05 vs. C/*CL*/-; ^&^ *p* < 0.05 vs. C/-/OVA; *p < 0.05 vs. fresh Treg cells.

We assessed Treg (CD4^+^CD25^+^FOXP3^+^) levels to observe inhibition of inflammation in fed-*CL* mice. Treg numbers and frequency in C/*CL*/- mice MLN were increased compared with that of C/-/- mice (Fig 4A–4C). Conversely, Treg levels were significantly decreased in C/-/OVA mice; that of C/*CL*/OVA mice were significantly increased compared with that of C/-/OVA mice (Fig 4A–4C). RT-PCR investigation of the expression of the Treg-specific transcription factor FOXP3 in whole lung tissue revealed increased *Foxp3* expression in both fed-*CL* groups relative to C/-/-. *Foxp3* expression was increased in C/*CL*/OVA mice as compared to C/-/OVA mice (Fig 4D). We performed a standard *in vitro* T cell inhibition assay to define the function of the fed-*CL*–induced Treg cells. Fresh CD4^+^CD25^+^ Treg cells and CD4^+^CD25^–^ T cells were obtained from 6–8-week-old untreated BALB/c mice; induced CD4^+^CD25^+^ Treg cells were obtained from C/*CL*/- mice. In comparison to the fresh Treg cells, the induced Treg cells had significantly improved inhibition function. In addition, the relative increase in CD4^+^CD25^+^ Treg cells was accompanied by increased proliferation inhibition function; the inhibition was strongest at a 1:1 ratio (Fig 4E). As expected, analysis of the cytokine content of the cell-free BALF revealed significantly decreased Treg cytokines (IL-10, TGF-β1) in C/-/OVA mice as compared with the C/-/- control. However, IL-10 and TGF-β1 were significantly higher in C/*CL*/OVA mice as compared with C/-/OVA mice (Fig 4F and 4G).

### Early-life fed-*CL* has an immunosuppressive effect

**Fig 5:**
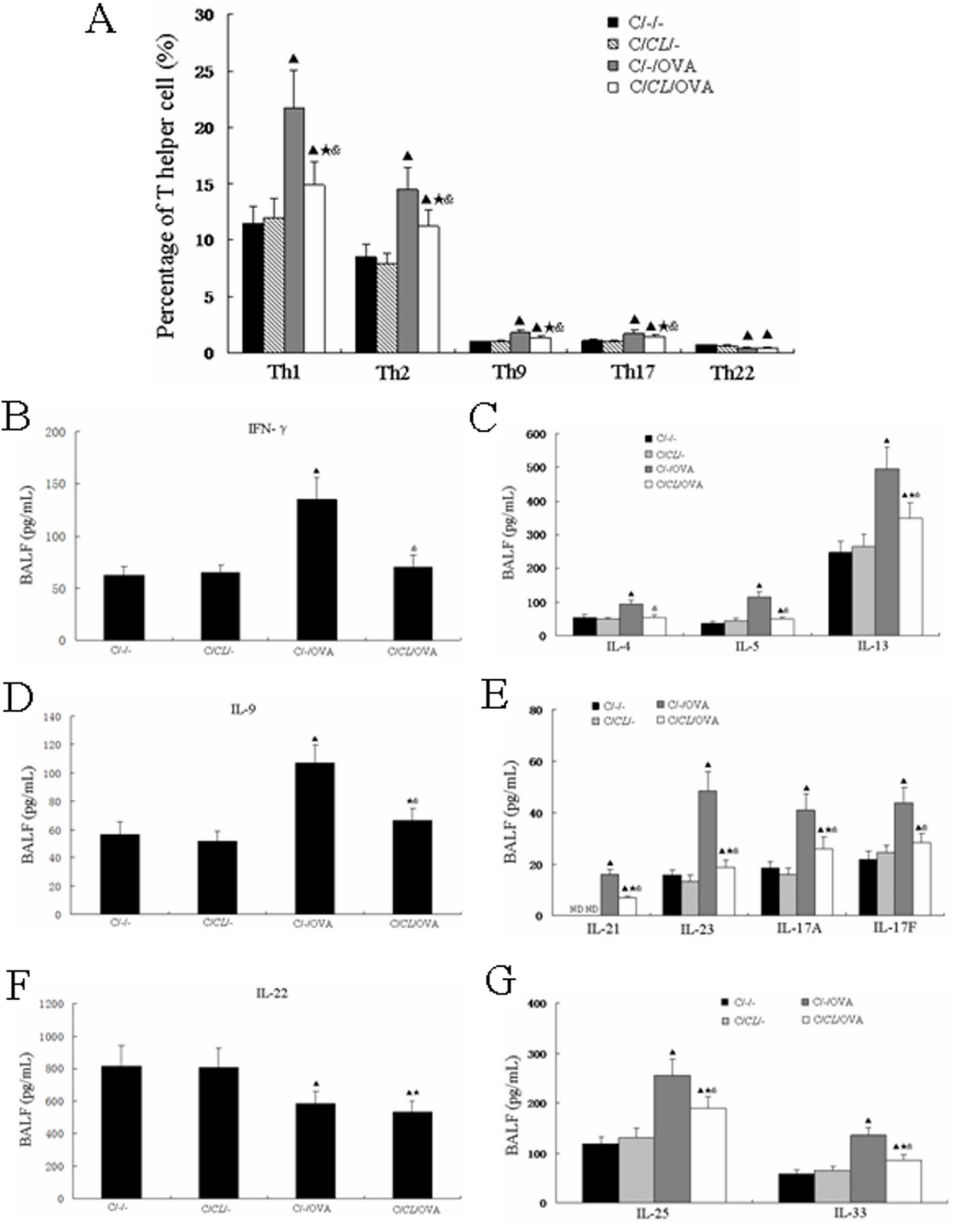
Early-life fed-*CL* had an immunosuppressive effect. (A) MLN T helper cell subset levels. ELISA determination of (B) Th1, (C) Th2, (D) Th9, (E) Th17, (F) Th22 cytokines, and (G) IL-25 and IL-33 protein levels in BALF. Data are plotted as the means ± SD. ^▲^ *p* < 0.05 vs. C/-/-; ^★^ *p* < 0.05 vs. C/*CL*/-; ^&^ *p* < 0.05 vs. C/-/OVA.

Th1, Th2, Th9, Th17, and Th22 lymphocytes are regularly associated with allergic asthma. Therefore, we assessed the levels of these lymphocytes to study the effects of fed-*CL* on Th1, Th2, Th9, Th17, and Th22 inflammation in a mouse asthma model. Th1, Th2, Th9, and Th17 were increased in C/-/OVA mice as compared to the control C/-/- mice, but were statistically decreased in C/*CL*/OVA mice as compared with C/-/OVA mice (*p* < 0.05, Fig 5A). Nevertheless, Th1, Th2, Th9, and Th17 levels were all significantly higher in C/*CL*/OVA mice as compared with C/-/- and C/*CL*/- mice (Fig 5A). Surprisingly, Th22 levels in C/-/OVA mice were significantly decreased as compared with the C/-/- mice; there were no significant differences between C/-/OVA and C/*CL*/OVA mice (Fig 5A). As expected, the cytokines of Th1 (IFN-γ), Th2 (IL-4, IL-5, IL-13), Th9 (IL-9), and Th17 (IL-17A, IL-17F, IL-21, IL-23) were all significantly increased in C/-/OVA mice as compared with the C/-/- mice (Fig 5B–5E); however, the levels were all significantly lower in C/*CL*/OVA mice as compared with C/-/OVA mice (Fig 5B–5E). The Th22 cytokine IL-22 in C/-/OVA mice was significantly decreased as compared with the C/-/- mice; there was no difference in IL-22 production in C/*CL*/OVA mice as compared with C/-/OVA mice (Fig 5F). Furthermore, IL-5, IL-13, IL-17A, IL-17F, IL-21, and IL-23 levels in C/*CL*/OVA mice were higher than that in C/-/- mice, and IL-13, IL-9, IL-17A, IL-21, and IL-23 levels in C/*CL*/OVA mice were higher than that in C/*CL*/- mice (Fig 5C–5E). IL-25 and IL-33 were recently recognized as being central to immune deviation toward pathogenic Th2 responses during T helper cell evolution [4]. In our study, IL-25 and IL-33 were statistically increased in both C/-/OVA and C/CL/OVA mice as compared with the C/-/- mice, and IL-25 and IL-33 levels were significantly lower in C/CL/OVA mice as compared with C/-/OVA mice (Fig 5G).

## Discussion

There are positive correlations between CD and the occurrence of asthma that presents during childhood and lasts into adulthood [31]. The rate of childhood asthma has increased over recent decades, particularly in the CD population [32]; this observation is supported by human epidemiological studies showing that the incidence of asthma is lower in individuals exposed to a greater diversity of bacteria at birth, i.e., vaginal vs. caesarian [21,33]. Indeed, there has been a progressive increase in the rates of CD worldwide, which the World Health Organization has reported as almost doubling in the last decade [34]. Several epidemiological studies have suggested that asthma risk is increased in children born by caesarian section [35–37]. A meta-analysis of 23 epidemiological studies concluded that asthma risk is 20% greater in children born by caesarian section as compared to those born vaginally [34]. A recent cohort study involving >37,000 Norwegian children concluded that children born by caesarian section have significantly increased asthma risk when they are 36 months old [38].

In the presence of the commensal microbiota, Treg cells are abundant in the intestinal tract, but are significantly decreased in germ-free animals. Treg numbers are normalized when the microbiota is reconstituted in germ-free mice, and a mixture of clostridial species (cluster IV and XIVa strains) appears to drive their development [39]. *Clostridium*-mediated induction of Treg cells in the colon may be responsible for systemic immune responses. In our study, a substantial increase in Treg cells was observed in mouse liver, lung, and spleen within three weeks of *Clostridium* inoculation. This finding suggests that *Clostridium* colonization in the intestine also affects the extraintestinal immune status [40]. In addition, the altered Schaedler flora, a defined group of eight bacterial strains that are common colonizers of mouse intestines and that include a combination of *Lactobacillus*, *Bacteroides*, *Flexistipes*, and *Clostridium* species, can be used to reconstitute the microbiota to re-establish Treg numbers [41,42]. Treg loss or absence results in the development of asthma, which adoptive transfer of natural or induced Treg cells can ameliorate [43]. Despite infants and young children representing a population highly affected by both *C*. *leptum* and asthma, there are very few data on the relationship between the two in the early stages of asthma development [40].

Allergic asthma is a chronic airway inflammation disease. In most asthma phenotypes, there are increased levels of eosinophils, neutrophils, and other inflammatory cells in the tissues, blood, and BALF. Compared with the control, there were increased total cell number, eosinophils, neutrophils, lymphocytes, and macrophages in the BALF of OVA-sensitized mice. Furthermore, there was aggravated inflammatory cell infiltration in lung tissues. We also observed persistent AHR. Asthma is defined as a variable level of airway obstruction typically accompanied by AHR; we measured airway resistance to monitor the effects of *C*. *leptum* on AHR, and found that *C*. *leptum* inhibited the OVA-induced AHR in response to the inhaled methacholine. The recruitment mechanisms of inflammatory cells associated with and presumably causing AHR are well-studied. Inflammatory cell migration in to the lung, specifically, that of eosinophils, lymphocytes, and neutrophils, is a major contributor to the development of allergic airway inflammation. Increased eosinophil numbers in the BALF and lung inflammatory infiltrates is a characteristic of asthma. Our results clearly demonstrate that *C*. *leptum* significantly reduces eosinophils, lymphocytes, and neutrophils in BALF and airway tissue inflammatory infiltrates.

In this study, we demonstrated an immunosuppressive environment in the lung in response to early-life fed-*CL*. Feeding weanling mice with *C*. *leptum* increased MLN CD4^+^CD25^+^FOXP3^+^ Treg frequency and number. Additionally, there were decreased Treg percentages in the lungs of OVA-sensitized mice. Quantitative and functional impairment of pulmonary CD4^+^CD25^+^Foxp^+^ Treg cells in pediatric asthma patients has been observed, demonstrating that CD4^+^CD25^+^FOXP3^+^ Treg cells can reverse established allergic airway inflammation and prevent airway remodeling [44]. These findings all suggest that decreased IL-10 and TGF-β production causes impaired function of the local airway CD4^+^CD25^+^FOXP3^+^ Treg cells in this mouse model of asthma. As Treg cells inhibit disease-promoting immune responses, enhancing Treg function is an attractive potential therapy for treating allergic asthma. We also show that early-life fed-*CL* increased Treg numbers in the target tissue–associated lymph nodes, *Foxp3* expression in the lung, and IL-10 and TGF-β1 levels in BALF. This immunosuppression could have beneficial effects with respect to asthma airway inflammation.

Indeed, our data demonstrate alleviated allergic responses in mice that had been fed with *C*. *leptum* as infants and then challenged with OVA during weaning. This was evidenced by AHR and the decreased Th1, Th2, and Th17 cell numbers in MLN and related cytokines (IFN-γ, IL-4, IL-5, IL-13, IL-17A, IL-17F, IL-21, IL-23) in the OVA-sensitized fed-*CL* adult mice. Interestingly, OVA-challenged adult mice demonstrate a Th9-mediated response [44]; we found that OVA challenge in infant mice also featured a dominant Th9 component. Therefore, it is of little surprise that fed-*CL* plus OVA challenge inhibited the Th9 response as compared with OVA challenge alone. Th22 cells are recent siblings of Th17 cells that predominantly produce IL-22 and represent a separate T helper subset with distinct gene expression and functions. Recent evidence indicates that IL-22 plays an important role in the pathogenesis of autoimmune diseases, including psoriasis, systemic lupus erythematosus, multiple sclerosis, rheumatoid arthritis, and allergic diseases, implicating Th22 cells and IL-22 as potential therapeutic targets in autoimmune diseases [11,45]. Furthermore, researchers have only begun to explore the role of IL-22 in allergy and asthma, but the effect of IL-22 is inconclusive. IL-22 attenuates the allergic response in the lungs of mice, demonstrating the negative regulatory function of IL-22 in allergy [46]. IL-22 also appears important in the defense against severe chronic rhinosinusitis, which often develops from allergic rhinitis and is associated with IL-22 receptor polymorphisms [47]. Here, we show that OVA significantly decreases MLN Th22 frequency and BALF IL-22 levels and that fed-*CL* does not affect Th22 frequency and IL-22. Furthermore, previous studies found that B-cell expansion is required to induce Treg generation and T-cell tolerance. Consequently, it has been speculated that B-cells play a role in the Treg increase in mice with early-life *C*. *leptum* exposure [48,49].

In conclusion, we demonstrate that the immunosuppressive effects of fed-*CL* in infant mice are partly due to the promotion of Treg differentiation. The presence of this inhibitory environment attenuated OVA-specific responses in the lung, leading to decreased overall inflammation and AHR. These findings illustrate a negative regulatory mechanism for asthma development in the context of *C*. *leptum* with OVA during early life, illustrating the importance of *C*. *leptum* in alleviating early-life asthma development.

## References

[1] Bihouée T, Bouchaud G, Chesné J, Lair D, Rolland-Debord C, Braza F, et al Food allergy enhances allergic asthma in mice. Respir Res. 2014; 15: 142 10.1186/s12931-014-0142-x 25433406 PMC4255648

[2] Takemura M, Niimi A, Matsumoto H, Ueda T, Matsuoka H, Yamaguchi M, et al Clinical, physiological and anti-inflammatory effect of montelukast in patients with cough variant asthma. Respiration. 2012; 83(4): 308–315. 10.1159/000332835 22094623

[3] Shurin MR, Yanamala N, Kisin ER, Tkach AV, Shurin GV, Murray AR, et al Graphene oxide attenuates Th2-type immune responses, but augments airway remodeling and hyperresponsiveness in a murine model of asthma. ACS Nano. 2014; 8(6): 5585–5599. 10.1021/nn406454u 24847914 PMC4072415

[4] Wisniewski JA, Borish L. Novel cytokines and cytokine-producing T cells in allergic disorders. Allergy Asthma Proc. 2011; 32(2): 83–94. 10.2500/aap.2011.32.3428 21439160

[5] Neu J, Rushing J. Cesarean versus vaginal delivery: long-term infant outcomes and the hygiene hypothesis. Clin Perinatol. 2011; 38(2): 321–331. 10.1016/j.clp.2011.03.008 21645799 PMC3110651

[6] Zhu J, Yamane H, Paul WE. Differentiation of effector CD4 T cell populations. Annu Rev Immunol. 2010; 28: 445–489. 10.1146/annurev-immunol-030409-101212 20192806 PMC3502616

[7] Wakashin H, Hirose K, Maezawa Y, Kagami S, Suto A, Watanabe N, et al IL-23 and Th17 cells enhance Th2-cell‑mediated eosinophilic airway inflammation in mice. Am J Respir Crit Care Med. 2008; 178(10): 1023–1032. 10.1164/rccm.200801-086OC 18787221

[8] Mitsdoerffer M, Lee Y, Jäger A, Kim HJ, Korn T, Kolls JK, et al Proinflammatory T helper type 17 cells are effective B-cell helpers. 2010; 107(32):14292–14297. 10.1073/pnas.1009234107 20660725 PMC2922571

[9] Xing J, Wu Y, Ni B. Th9: a new player in asthma pathogenesis? J Asthma J Asthma. 2011; 48(2):115–125. 10.3109/02770903.2011.554944 21294663

[10] Temann UA, Ray P, Flavell RA. Pulmonary overexpression of IL-9 induces Th2 cytokine expression, leading to immune pathology. J Clin Invest. 2002; 109(1):29–39. 11781348 10.1172/JCI13696 PMC150821

[11] Raphael I, Nalawade S, Eagar TN, Forsthuber TG. T cell subsets and their signature cytokines in autoimmune and inflammatory diseases. Cytokine. 2015; 74(1): 5–17. 10.1016/j.cyto.2014.09.011 25458968 PMC4416069

[12] Li YN, Huang F, Cheng HJ, Li SY, Liu L, Wang LY. Intestine-derived Clostridium leptum induces murine tolerogenic dendritic cells and regulatory T cells in vitro. Hum Immunol. 2014; 75(12): 1232–1238. 10.1016/j.humimm.2014.09.017 25300998

[13] Gavin MA, Rasmussen JP, Fontenot JD, Vasta V, Manganiello VC, Beavo JA, et al Foxp3-dependent programme of regulatory T-cell differentiation. Nature. 2007; 445(7129):771–775. 17220874 10.1038/nature05543

[14] Wu Y, Borde M, Heissmeyer V, Feuerer M, Lapan AD, Stroud JC, et al FOXP3 controls regulatory T cell function through cooperation with NFAT. Cell. 2006; 126(2): 375–387. 16873067 10.1016/j.cell.2006.05.042

[15] Zheng Y, Josefowicz SZ, Kas A, Chu TT, Gavin MA, Rudensky AY. Genome-wide analysis of Foxp3 target genes in developing and mature regulatory T cells. Nature. 2007; 445(7130): 936–940. 17237761 10.1038/nature05563

[16] Tamachi T, Maezawa Y, Ikeda K, Kagami S, Hatano M, Seto Y, et al IL-25 enhances allergic airway inflammation by amplifying a TH2 cell-dependent pathway in mice. J Allergy Clin Immunol. 2006; 118(3): 606–614. 16950278 10.1016/j.jaci.2006.04.051

[17] Biasucci G, Benenati B, Morelli L, Bessi E, Boehm G. Cesarean delivery may affect the early biodiversity of intestinal bacteria. J Nutr. 2008; 138(9):1796S–1800S. 18716189 10.1093/jn/138.9.1796S

[18] Debley JS, Smith JM, Redding GJ, Critchlow CW. Childhood asthma hospitalization risk after cesarean delivery in former term and premature infants. Ann Allergy Asthma Immunol. 2005; 94(2): 228–233. 15765737 10.1016/S1081-1206(10)61300-2

[19] Grönlund MM, Lehtonen OP, Eerola E, Kero P. Fecal microflora in healthy infants born by different methods of delivery: permanent changes in intestinal flora after cesarean delivery. J Pediatr Gastroenterol Nutr. 1999; 28(1): 19–25. 9890463 10.1097/00005176-199901000-00007

[20] Ganguli K, Walker WA. Probiotics in the prevention of necrotizing enterocolitis. J Clin Gastroenterol. J Clin Gastroenterol. 2011; 45 Suppl: S133–138. 10.1097/MCG.0b013e318228b799 21992952

[21] Collado MC, Rautava S, Isolauri E, Salminen S3. Gut microbiota: a source of novel tools to reduce the risk of human disease? Pediatr Res. 2015; 77(1–2): 182–188. 10.1038/pr.2014.173 25335085

[22] Li YN, Huang F, Liu L, Qiao HM, Li Y, Cheng HJ. Effect of oral feeding with Clostridium leptum on regulatory T-cell responses and allergic airway inflammation in mice. Ann Allergy Asthma Immunol. 2012; 109(3): 201–207. 10.1016/j.anai.2012.06.017 22920076

[23] Sghir A, Gramet G, Suau A, Rochet V, Pochart P, Dore J. Quantification of bacterial groups within human fecal flora by oligonucleotide probe hybridization. Appl Environ Microbiol. 2000; 66(5): 2263–2266. 10788414 10.1128/aem.66.5.2263-2266.2000 PMC101487

[24] Gunzer M, Weishaupt C, Planelles L, Grabbe S. Two-step negative enrichment of CD4+ and CD8+ T cells from murine spleen via nylon wool adherence and an optimized antibody cocktail. J Immunol Methods. 2001; 258(1–2): 55–63. 11684123 10.1016/s0022-1759(01)00466-5

[25] Bhattacharya P, Fan J, Haddad C, Essani A, Gopisetty A, Elshabrawy HA, et al A novel pancreatic β-cell targeting bispecific-antibody (BsAb) can prevent the development of type 1 diabetes in NOD mice. Clin Immunol. 2014; 153(1): 187–198. 10.1016/j.clim.2014.04.014 24792135 PMC4077286

[26] Chen Z, Kim SJ, Chamberlain ND, Pickens SR, Volin MV, Volkov S, et al. The novel role of IL-7 ligation to IL-7 receptor in myeloid cells of rheumatoid arthritis and collagen-induced arthritis. 2013; 190(10): 5256–5266. 10.4049/jimmunol.1201675 PMC3686279 23606539

[27] Livak KJ, Schmittgen TD. Analysis of relative gene expression data using real-time quantitative PCR and the 2(-Delta Delta C(T)) method. Methods. 2001; 25(4): 402–408. 11846609 10.1006/meth.2001.1262

[28] Elshabrawy HA, Coughlin MM, Baker SC, Prabhakar BS. Human monoclonal antibodies against highly conserved HR1 and HR2 domains of the SARS-CoV spike protein are more broadly neutralizing. PLoS One. 2012; 7(11):e50366 10.1371/journal.pone.0050366 23185609 PMC3503966

[29] Elshabrawy HA, Fan J, Haddad CS, Ratia K, Broder CC, Caffrey M, et al Identification of a broad-spectrum antiviral small molecule against severe acute respiratory syndrome coronavirus and Ebola, Hendra, and Nipah viruses by using a novel high-throughput screening assay. J Virol. 2014; 88(8):4353–4365. 10.1128/JVI.03050-13 24501399 PMC3993759

[30] Ma C, Ma Z, Fu Q, Ma S. Anti-asthmatic effects of baicalin in a mouse model of allergic asthma. Phytother Res. 2014; 28(2): 231–237. 10.1002/ptr.4983 23580257

[31] Hyde MJ, Modi N. The long-term effects of birth by caesarean section: the case for a randomised controlled trial. Early Hum Dev. 2012; 88(12):943–949. 10.1016/j.earlhumdev.2012.09.006 23036493

[32] Thavagnanam S, Fleming J, Bromley A, Shields MD, Cardwell CR. A meta-analysis of the association between Caesarean section and childhood asthma. Clin Exp Allergy. 2008; 38(4):629–633. 10.1111/j.1365-2222.2007.02780.x 18352976

[33] Van Nimwegen FA, Penders J, Stobberingh EE, Postma DS, Koppelman GH, Kerkhof M. Mode and place of delivery, gastrointestinal microbiota, and their influence on asthma and atopy. J Allergy Clin Immunol. 2011; 128(5):948–955.e1-3. 10.1016/j.jaci.2011.07.027 21872915

[34] Thavagnanam S, Fleming J, Bromley A, Shields MD, Cardwell CR. A meta analysis of the association between Caesarean section and childhood asthma. Clin Exp Allergy. 2008; 38(4):629–633. 10.1111/j.1365-2222.2007.02780.x 18352976

[35] Decker E, Engelmann G, Findeisen A, Gerner P, Laass M, Ney D, Cesarean delivery is associated with celiac disease but not inflammatory bowel disease in children. Pediatrics. 2010; 125(6):e1433–1440. 10.1542/peds.2009-2260 20478942

[36] Barros FC, Matijasevich A, Hallal PC, Horta BL, Barros AJ, Menezes AB, et al Cesarean section and risk of obesity in childhood, adolescence, and early adulthood: evidence from 3 Brazilian birth cohorts. Am J Clin Nutr. 2012; 95(2):465–470. 10.3945/ajcn.111.026401 22237058 PMC3260073

[37] Magnus MC, Håberg SE, Stigum H, Nafstad P, London SJ, Vangen S. Delivery by Cesarean section and early childhood respiratory symptoms and disorders: the Norwegian mother and child cohort study. Am J Epidemiol. 2011; 174(11):1275–1285. 10.1093/aje/kwr242 22038100 PMC3254156

[38] Atarashi K, Tanoue T, Oshima K, Suda W, Nagano Y, Nishikawa H, et al Treg induction by a rationally selected mixture of Clostridia strains from the human microbiota. Nature. 2013; 500(7461):232–236. 10.1038/nature12331 23842501

[39] Round JL, Mazmanian SK. Inducible Foxp3+ regulatory T-cell development by a commensal bacterium of the intestinal microbiota. Proc Natl Acad Sci U S A. 2010; 107(27):12204–12209. 10.1073/pnas.0909122107 20566854 PMC2901479

[40] Tanoue T, Shima T, Imaoka A, Kuwahara T, Momose Y, Cheng G, et al Induction of colonic regulatory T cells by indigenous Clostridium species. Science. 2011; 331(6015):337–341. 10.1126/science.1198469 21205640 PMC3969237

[41] Round JL, Lee SM, Li J, Tran G, Jabri B, Chatila TA, et al The Toll-like receptor 2 pathway establishes colonization by a commensal of the human microbiota. Science. 2011; 332(6032):974–977. 10.1126/science.1206095 21512004 PMC3164325

[42] Salzman NH. The role of the microbiome in immune cell development. Ann Allergy Asthma Immunol. 2014; 113(6): 593–598. 10.1016/j.anai.2014.08.020 25466801

[43] Hartl D, Koller B, Mehlhorn AT, Reinhardt D, Nicolai T, Schendel DJ, et al Quantitative and functional impairment of pulmonary CD4+CD25hi regulatory T cells in pediatric asthma. J Allergy Clin Immunol. 2007; 119(5): 1258–1266. 17412402 10.1016/j.jaci.2007.02.023

[44] Horka H, Staudt V, Klein M, Taube C, Reuter S, Dehzad N, et al The tick salivary protein sialostatin L inhibits the Th9-derived production of the asthma-promoting cytokine IL-9 and is effective in the prevention of experimental asthma. J Immunol. 2012; 188(6):2669–2676. 10.4049/jimmunol.1100529 22327077 PMC3523721

[45] Tian T, Yu S, Ma D. Th22 and related cytokines in inflammatory and autoimmune diseases. Expert Opin Ther Targets. 2013; 17(2): 113–125. 10.1517/14728222.2013.736497 23256771

[46] Schnyder B, Lima C, Schnyder-Candrian S. Interleukin-22 is a negative regulator of the allergic response. Cytokine. 2010; 50(2): 220–227. 10.1016/j.cyto.2010.02.003 20194033

[47] Smith AJ, Humphries SE. Cytokine and cytokine receptor gene polymorphisms and their functionality. Cytokine Growth Factor Rev. 2009; 20(1):43–59. 10.1016/j.cytogfr.2008.11.006 19038572

[48] Ashour HM, Niederkorn JY. Expansion of B cells is necessary for the induction of T-cell tolerance elicited through the anterior chamber of the eye. Int Arch Allergy Immunol. 2007; 144(4):343–346. 17671393 10.1159/000106461

[49] Ashour HM, Seif TM. The role of B cells in the induction of peripheral T cell tolerance. J Leukoc Biol. 2007; 82(5):1033–1039. 17656652 10.1189/jlb.0507310

